# The Elias University Hospital Approach: A Visual Guide to Ultrasound-Guided Botulinum Toxin Injection in Spasticity: Part II—Proximal Upper Limb Muscles

**DOI:** 10.3390/toxins17060276

**Published:** 2025-05-31

**Authors:** Marius Nicolae Popescu, Claudiu Căpeț, Cristina Beiu, Mihai Berteanu

**Affiliations:** 1 Department of Physical and Rehabilitation Medicine, Elias Emergency University Hospital, Carol Davila University of Medicine and Pharmacy, 020021 Bucharest, Romania; marius.popescu@umfcd.ro (M.N.P.); mberteanu@gmail.com (M.B.); 2 Clinic of Physical and Rehabilitation Medicine, Elias Emergency University Hospital, 011461 Bucharest, Romania; claudiu.capet@gmail.com; 3 Department of Oncologic Dermatology, Elias Emergency University Hospital, Carol Davila University of Medicine and Pharmacy, 020021 Bucharest, Romania

**Keywords:** post-stroke spasticity, botulinum toxin-A injections, ultrasound-guided therapy, proximal upper limb muscles, musculoskeletal ultrasound

## Abstract

Ultrasound-guided botulinum toxin type A (BoNT-A) injections play a critical role in the management of upper limb spasticity. This is the second part of ‘The Elias University Hospital Approach: A Visual Guide to Ultrasound-Guided Botulinum Toxin Injection in Spasticity’ and it focuses on the proximal upper limb muscles, complementing the first part, which addressed the distal upper limb muscles. This guide provides a detailed analysis of ultrasound anatomy, clinical relevance, and injection strategies for the latissimus dorsi, teres major, subscapularis, pectoralis major, pectoralis minor, deltoid, triceps brachii, biceps brachii, brachialis, and brachioradialis. Using the Elias University Hospital (EUH) model, it presents a structured approach to BoNT-A administration, ensuring precision, safety, and optimal outcomes in spasticity management. To enhance clinical application, this guide incorporates a wide array of high-quality ultrasound images and dynamic videos, offering a comprehensive and practical understanding of scanning techniques, anatomical structures, and injection procedures. This second part of the series serves as an essential reference for clinicians, aligning with the first installment to provide a complete and systematic approach to ultrasound-guided BoNT-A therapy for upper limb spasticity.

## 1. Introduction

Upper limb spasticity, commonly occurring after stroke or other neurological injuries, poses significant challenges to mobility and quality of life. Botulinum toxin type A (BoNT-A) injections are widely used in managing spasticity, helping to reduce muscle overactivity and improve functional outcomes. Accurate identification of injection sites is crucial for achieving therapeutic success and minimizing risks [1].

While several atlases and guides have advanced the field with valuable anatomical and technical insights into ultrasound-guided BoNT-A injections [2,3,4,5], most resources provide broad anatomical overviews or focus on limited muscle groups. The Elias University Hospital (EUH) approach distinguishes itself by integrating detailed sonographic anatomy, dynamic muscle evaluation, and practical injection techniques tailored to the management of proximal upper limb spasticity. This includes the identification of intramuscular and intermuscular fascial planes, precise localization of motor points, and real-time ultrasound imaging to guide injections. Our approach combines established anatomical knowledge with clinical insights we developed over years and serves as a practical resource for clinicians aiming to enhance precision and efficacy in BoNT-A administration for spasticity management. In this way, it complements and extends the current literature by offering a practical, integrated framework that addresses the specific needs of clinicians treating spasticity in complex upper limb presentations.

While the first part of this series provided a visual guide to ultrasound-guided BoNT-A injections for distal upper limb muscles [6], this second part focuses on the proximal upper limb muscles, which play a central role in shoulder, arm, and forearm movements. These muscles include the latissimus dorsi, teres major, subscapularis, pectoralis major, pectoralis minor, deltoid, triceps brachii, biceps brachii, brachialis, and brachioradialis.

## 2. Proximal Upper Limb Muscles Implicated in Post-Stroke Spasticity

### 2.1. Latissimus Dorsi (LD)

#### 2.1.1. Overview

The latissimus dorsi (LD) is one of the muscles frequently targeted in spastic patterns of the upper limb, which include resistance to passive movement during maneuvers of flexion, abduction, and lateral rotation of the humerus at the shoulder joint [7].

#### 2.1.2. Ultrasound Identification

In our clinical practice, the LD is identified using ultrasound by placing the transducer transversely on the posterior surface of the trunk, approximately 9 cm distal to the inferior angle of the scapula. Superficial to the rib cortex and intercostal muscles, the LD appears clearly defined [8].

#### 2.1.3. Key Ultrasound Landmarks

**Figure 1:**
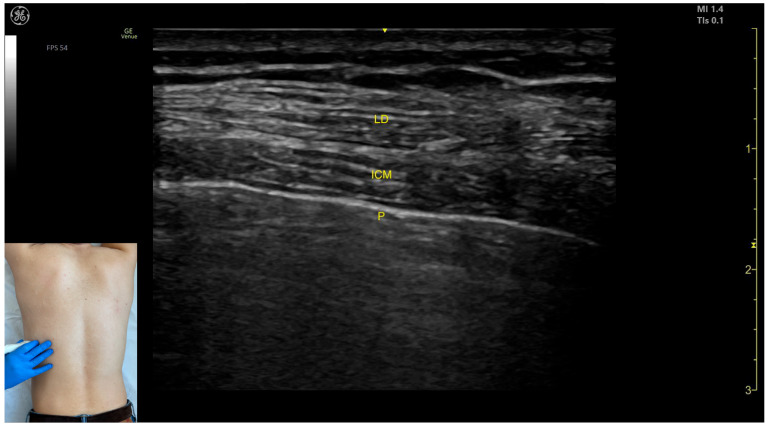
Ultrasound anatomy of the latissimus dorsi (LD) with key landmarks: LD—latissimus dorsi; ICM—intercostal muscle; and P—pleura.

**Figure 2:**
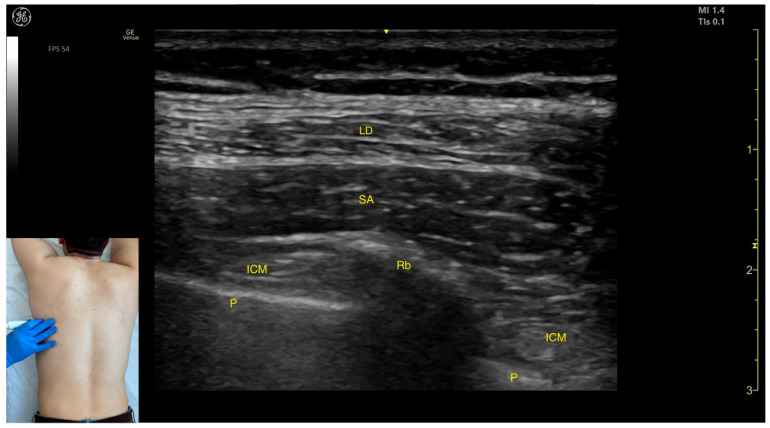
Ultrasound anatomy of the latissimus dorsi (LD) and serratus anterior (SA) with key landmarks: LD—latissimus dorsi; SA—serratus anterior; ICM—intercostal muscle; Rb—rib; and P—pleura.

The key ultrasound landmarks include the following (Figure 1 and Figure 2) [9,10]:

- Muscle position: The LD is the most superficial muscle mass on the posterior surface of the trunk at this level.
- Muscle morphology: It has a more pronounced fascia that separates it from the subcutaneous plane than the fascia that separates it from the intercostal muscle (ICM) muscle during BoNT-A injections.
- Dynamic evaluation: Scanning proximally (~5 cm) shows a reduction in the size of the LD and the appearance and enlargement of the serratus anterior (SA) muscle beneath it. Contraction is visible with extension, adduction, and medial rotation maneuvers of the humerus at the shoulder joint [7]. At this level the LD has a more pronounced fascia that separates it from the subcutaneous plane and the SA muscle.

#### 2.1.4. Clinical Implications and Injection Strategy

The LD is frequently injected when treating spastic patterns that involve adduction, extension, and internal rotation of the shoulder joint, often in combination with the teres major muscle [11].

Due to the large size of the LD, multiple injection sites are typically required. To reduce the total dosage of BoNT-A and improve the management of spasticity, the injections should be administered in areas with the highest density of neural divisions. If these optimal zones are not targeted, higher doses of BoNT-A are needed to improve the upper limb’s range of motion, which increases the risk of antibody formation and reduces the treatment’s effectiveness [10].

According to a 2022 study aimed at determining the regions with the greatest neural arborization in the LD, the muscle was divided into segments of 10% along a line extending from the spinous process of vertebra T5 to the midpoint of the iliac crest (0%) and continuing to the medial aspect of the surgical neck of the humerus (100%). The muscle was further subdivided into equal-width medial, middle, and lateral portions. The regions with the highest neural arborization were found to be between 40 and 60% in the medial and lateral parts, between 30 and 60% in the middle part, and between 70 and 90% across the medial, middle, and lateral regions [10].

In our clinical practice, the preferred area for local BoNT-A injections is the point of maximum muscle thickness identified via US, with the transducer placed transversely on the posterior surface of the trunk, approximately 7 cm distal to the inferior angle of the scapula and along the interscapulovertebral line corresponding to the T7-L5 vertebrae. The use of musculoskeletal US in approaching the LD allows precise placement of the substance at the target site and prevents the occurrence of pneumothorax.

### 2.2. Teres Major (TM)

#### 2.2.1. Overview

The teres major (TM) is one of the muscles frequently targeted in spastic patterns of the upper limb that involve resistance to passive movement during maneuvers of abduction and lateral rotation of the humerus at the shoulder joint [12].

#### 2.2.2. Ultrasound Identification

In our clinical practice, with the patient’s shoulder held in adduction and internal rotation, the TM is identified via US by placing the transducer transversely on the posterior surface of the thoracic wall, approximately 2–3 cm distal to the posterior axillary fold. Superficial to the rib cortex and intercostal muscles, three muscle masses are identified from medial to lateral: the subscapularis muscle, the TM muscle, and the LD muscle.

#### 2.2.3. Key Ultrasound Landmarks

**Figure 3:**
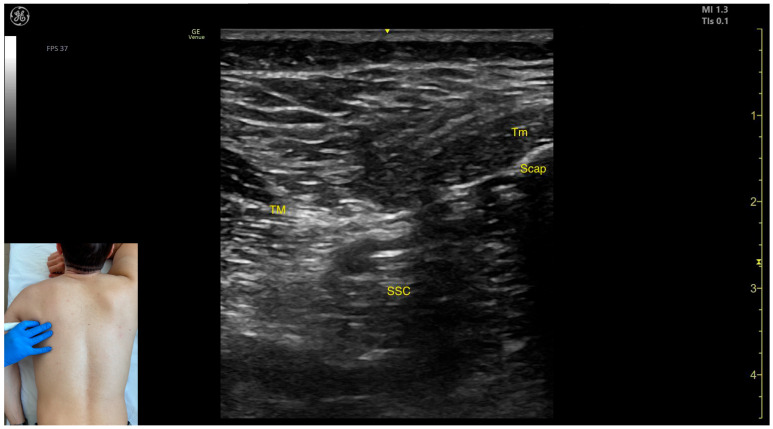
Ultrasound anatomy of the teres major (TM) with key landmarks: TM—teres major; Tm—teres minor; SSC—subscapularis; and Scap—scapula.

The key ultrasound features include the following (Figure 3) [13,14]:

- Muscle morphology: The TM is a large muscle, visible from the depth of the rib cortex and intercostal muscles up to the subcutaneous plane.
- Muscle position: Medially and superficially, the teres minor (Tm) muscle is observed; medially and deeply, the subscapularis muscle and scapular cortex are visible; while laterally and deeply, the LD muscle is identified.
- External fascia: It has a pronounced fascia that separates it from adjacent muscle masses during BoNT-A injections.
- Dynamic evaluation: During dynamic evaluation, scanning proximally toward the axilla shows the transformation of the LD into a tendon, alongside which the TM forms the inferolateral portion of the posterior axillary wall [7]. The contraction of the TM is visible during adduction and internal rotation maneuvers of the humerus at the shoulder joint [12].

#### 2.2.4. Clinical Implications and Injection Strategy

The TM is a muscle frequently injected in spastic patterns involving abduction, elevation, flexion, and internal rotation of the shoulder joint, in combination with the pectoralis major and subscapularis muscles. It is also targeted in spastic patterns that include adduction, extension, and internal rotation of the shoulder joint, alongside the LD [11]. In addition to treating spasticity, local BoNT-A injections are also used to manage spastic hemiplegic shoulder pain involving internal rotation and adduction. For this purpose, both superficial muscles, such as the pectoralis major and LD, and deep muscles, like the TM and subscapularis, are injected [15,16,17].

According to a study by Yi et al. that aimed to determine the optimal injection site for BoNT-A in the TM muscle, the highest density of intramuscular nerve endings was identified in the middle 20% of the TM muscle from its origin to its insertion [18].

In our clinical practice, the preferred injection site for local BoNT-A administration is at the point of maximum muscle thickness identified through US with the transducer placed transversely on the posterior surface of the thoracic wall approximately 2–3 cm distal to the posterior axillary fold.

### 2.3. Subscapularis (SSC)

#### 2.3.1. Overview

The subscapularis (SSC) is one of the muscles frequently targeted in spastic patterns of the upper limb that involve resistance to passive movement during abduction and lateral rotation maneuvers of the humerus at the shoulder joint [12,19].

#### 2.3.2. Ultrasound Identification

The SSC can be identified via US in the posterior window with the shoulder positioned in flexion, abduction, and internal rotation. For this assessment, the transducer is placed transversely on the posterior surface of the thoracic wall, lateral to the lateral edge of the scapula, approximately 1 cm distal to the posterior axilla [5].

It can also be assessed through the anterior window with the arm positioned in adduction and external rotation at the shoulder joint, 90-degree flexion at the elbow, and the palm facing upward. The transducer is placed on the anterior surface of the arm, approximately 2–3 cm from the clavicle [20].

#### 2.3.3. Key Ultrasound Landmarks

**Figure 4:**
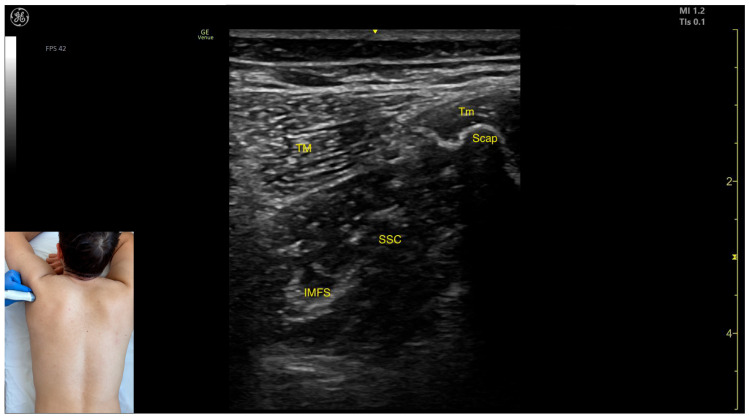
Ultrasound anatomy of the subscapularis (SSC) posterior view with key landmarks: SSC—subscapularis; TM—teres major; Tm—teres minor; Scap—scapula; and IMFS—intramuscular fascial septum.

The key ultrasound landmarks in the posterior window include the following (Figure 4) [21,22,23]:

- Muscle position: At this level, the SSC represents the first muscle mass located superficial to the ribs and intercostal muscles. Superficial to the SSC, the TM is observed laterally, and the Tm is observed medially.
- Intramuscular fascial septum: It features an intramuscular fascial septum (IMFS) that separates the muscle into superior and inferior compartments, which can be approached individually if needed.
- External fascia: Unlike other muscles, the SSC does not present a pronounced fascial layer that separates it from the TM during BoNT-A injections.
- Dynamic evaluation: During dynamic evaluation, visible muscle contraction is seen during internal rotation and adduction maneuvers of the humerus at the shoulder joint [12,19].

**Figure 5:**
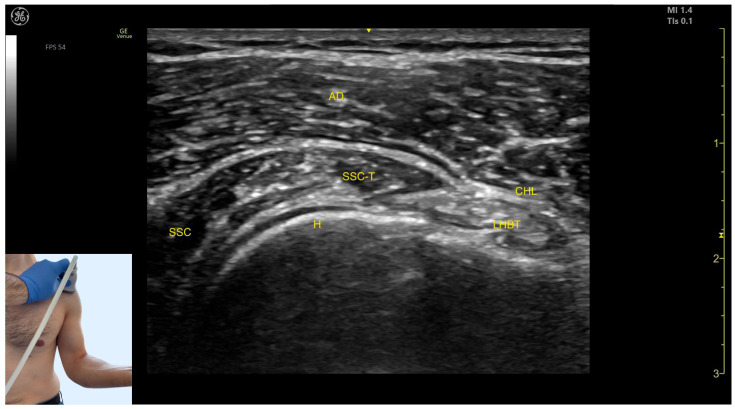
Ultrasound anatomy of the subscapularis (SSC) anterior view with key landmarks: AD—anterior deltoid; SSC—subscapularis; SSC-T—subscapularis tendon; H—humerus; LHBT—long head bicep tendon; and CHL—coracohumeral ligament.

The key ultrasound features in the anterior window include the following (Figure 5) [21,24]:

- Superficial to the humeral cortex, the long head tendon of the biceps brachii (LHBT) can be seen, encased by the coracohumeral ligament (CHL).
- Medially, the tendon of the SSC and the muscle itself are visible.
- The anterior deltoid muscle is located superficially over all these structures.

#### 2.3.4. Clinical Implications and Injection Strategy

According to specialized studies, the SSC and pectoralis major are the most frequently involved muscles in spastic patterns that include internal rotation and adduction of the shoulder joint [25,26]. Local BoNT-A injections into the SSC are effective not only for reducing spasticity by improving the range of motion but also for alleviating hemiplegic shoulder pain [26,27,28].

A 2023 study revealed that the intramuscular fascial septum of the SSC divides it into two compartments, each with distinct innervation zones and fiber compositions [23]:

- The superior compartment contains mainly type II fibers (fast-twitch). Along the muscle length—from the insertion on the lesser tubercle of the humerus to the origin at the subscapular fossa—the highest concentration of type I fibers (slow-twitch) is found around 22% of the distance from the insertion, while the type II fibers peak at approximately 77% of the muscle length, closer to the origin [21,23].
- The inferior compartment is composed predominantly of type I fibers (slow-twitch). The greatest density of type I fibers is located around 81% of the distance from the insertion to the origin, whereas type II fibers are most concentrated near 18% of the muscle length, closer to the insertion [21,23].

The intramuscular fascial septum acts as a substantial barrier to the diffusion of injected substances between the two compartments. This anatomical feature necessitates individual targeting of each compartment during BoNT-A injections to achieve optimal therapeutic outcomes [23].

In our clinical practice, the preferred injection site for local BoNT-A administration is at the point of maximum muscle thickness, as identified via US using the posterior window. This approach allows for the ultrasound-guided injection of type I fibers in the superior compartment and type II fibers in the inferior compartment. To effectively inject the type II fibers of the superior compartment and the type I fibers of the inferior compartment, a blind approach is often required through the posterior window via the inferior border of the scapula. For this technique, the needle is advanced deeply from the inferior angle of the scapula toward the medial third of the upper margin of the scapula.

The use of musculoskeletal US in the posterior window approach ensures precise placement of the toxin at the target site and helps prevent pneumothorax during the procedure. Based on our clinical experience, we typically do not use the anterior window for local BoNT-A injections. Not only is there often marked fibrosis, sarcomere loss, and a low density of motor endplates anteriorly, but patients requiring SSC injection frequently cannot achieve the degree of passive external rotation needed to access the anterior window. Consequently, the risk of muscle injury and suboptimal visualization outweighs the potential benefit of an anterior approach [29,30].

### 2.4. Pectoralis Major (Pmaj)

#### 2.4.1. Overview

The pectoralis major (Pmaj) is one of the muscles frequently targeted in spastic patterns of the upper limb that involve resistance to passive movement during abduction and lateral rotation maneuvers of the humerus at the shoulder joint [19].

#### 2.4.2. Ultrasound Identification

The Pmaj can be identified using musculoskeletal US by placing the transducer at a 45-degree angle toward the axilla on the anterior thoracic wall, approximately 2–3 cm distal to the lateral third of the clavicle. Superficial to the rib cortex and intercostal muscles, the following two muscle masses are observed [31]:

- The pectoralis minor (Pmin) muscle;
- The pectoralis major (Pmaj) muscle.

#### 2.4.3. Key Ultrasound Landmarks

**Figure 6:**
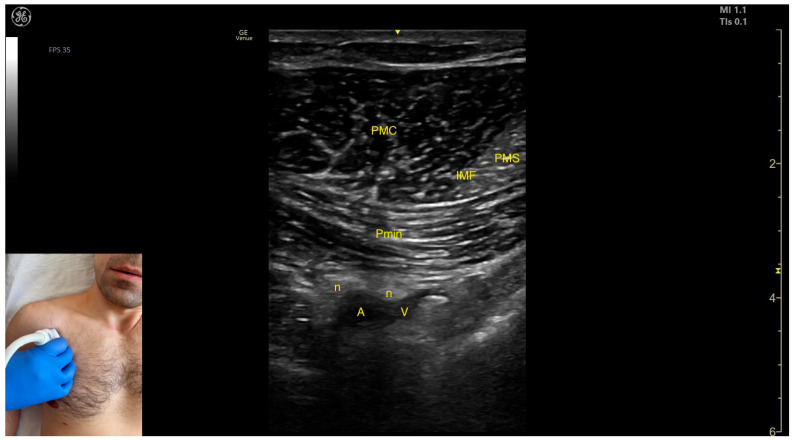
Ultrasound anatomy of the pectoralis major (Pmaj) with key landmarks: PMC—pectoralis major clavicular head; PMS—pectoralis major sternocostal head; IMF—intramuscular fascia; Pmin—pectoralis minor; n—nerve; A—artery; and V—vein.

The key ultrasound features include the following (Figure 6) [31,32,33]:

- Muscle position: It is the most superficial muscle mass on the anterior thoracic wall. Deep to the Pmaj, the following structures are observed: the pectoralis minor muscle, the axillary artery and vein, and distal nerve branches from the brachial plexus [32,33].
- Muscle morphology: The muscle has two heads that can be approached individually: the clavicular head (PMC), located laterally, and the sternocostal head (PMS), located medially. An intramuscular fascia (IMF) separates the two heads.
- External fascia: The Pmaj presents a pronounced fascia that clearly separates it from the subcutaneous plane and the Pmin during BoNT-A injections.
- Neurovascular landmark: The lateral pectoral nerve often courses alongside the thoracoacromial artery within the fascia separating P maj and P min. This can be targeted during diagnostic nerve blocks to distinguish reducible from non-reducible shoulder deformities in severe spasticity.
- Dynamic evaluation: During dynamic evaluation, scanning toward the humeral insertion reveals a gradual reduction in muscle thickness for both heads until they transform into tendons. Muscle contraction is visible during adduction and internal rotation maneuvers of the upper limb at the shoulder joint [19].

#### 2.4.4. Clinical Implications and Injection Strategy

The clavicular head of the Pmaj is responsible for flexion of the humerus, while the sternocostal head facilitates extension of the humerus from a flexed position. Together, these two heads produce strong adduction and medial rotation of the upper limb [19]. The Pmaj is the most frequently targeted muscle in spastic patterns that involve adduction and internal rotation of the shoulder, typically alongside the Pmin, LD, SSC, and TM [11,25,34]. Beyond its role in reducing resistance to passive movement and spasticity, BoNT-A injections into the Pmaj have proven effective in treating hemiplegic shoulder pain, both in the short and medium term. Notably, in the medium term, this approach is considered more effective than subscapular nerve block [34].

A 2021 study by Li et al. identified the densest intramuscular nerve regions of the Pmaj as being located at approximately 60% of the muscle length for the clavicular head and 55% of the muscle length for the sternocostal head, with both measurements taken from the origin to the insertion of the muscle [35].

In our clinical practice, the preferred injection zones for BoNT-A administration are at the points of maximum muscle thickness, identified via musculoskeletal US:

- For the clavicular head, the transducer is placed at a 45-degree angle toward the axilla, along the midclavicular line, approximately 2 cm from the clavicle.
- For the sternocostal head, the transducer is positioned longitudinally along the midclavicular line, about 5 cm distal to the clavicle.

### 2.5. Pectoralis Minor (Pmin)

#### 2.5.1. Overview

The pectoralis minor (Pmin) is a muscle targeted in spastic patterns of the upper limb that involve the posterior projection and elevation of the inferior angle of the scapula [7].

#### 2.5.2. Ultrasound Identification

The Pmin can be identified using musculoskeletal US by placing the transducer at a 45-degree angle toward the axilla on the anterior thoracic wall, along the midclavicular line, approximately 5–6 cm distal to the clavicle. Superficial to the rib cortex and intercostal muscles, the pectoralis minor muscle is visualized [36].

#### 2.5.3. Key Ultrasound Landmarks

**Figure 7:**
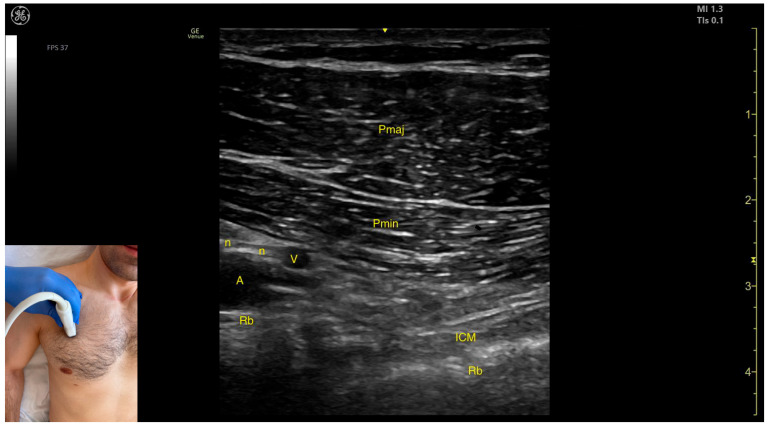
Ultrasound anatomy of the pectoralis minor (Pmin) with key landmarks: Pmaj—pectoralis major; Pmin—pectoralis minor; ICM—intercostal muscle; Rb—rib; n—nerve; A—artery; and V—vein.

The key ultrasound features include the following (Figure 7) [32,33,36]:

- Muscle position: It represents the first muscle mass located superficial to the rib cortex and intercostal muscles at this level. Superficial to the Pmin, the Pmaj muscle is observed. Deep to the Pmin, the axillary artery and vein, along with distal nerve branches from the brachial plexus, are visible.
- External fascia: The Pmin has a pronounced fascia that separates it from the Pmaj during BoNT-A injections.
- Dynamic evaluation: During dynamic evaluation, scanning proximally toward the coracoid process of the scapula shows a decrease in muscle thickness until the muscle transforms into a tendon [37].

#### 2.5.4. Clinical Implications and Injection Strategy

The Pmin is a muscle targeted in spastic patterns involving adduction and internal rotation of the shoulder joint, typically in conjunction with the Pmaj, LD, SSC, and TM [27]. Additionally, local BoNT-A injections can be used in the management of pectoralis minor syndrome, a condition characterized by hyperactivity of the Pmin resulting in compression of the underlying distal branches of the brachial plexus [32,33].

According to a 2023 study, the region with the highest density of intramuscular nerve arborization in the Pmin is located at three-quarters of the muscle length, measured from the origin to the insertion on the coracoid process of the scapula [38]. In our clinical practice, the preferred injection site for local BoNT-A administration is at the point of maximum muscle thickness, as identified via musculoskeletal US. The transducer should be placed lateralized at a 45-degree angle toward the axilla on the anterior thoracic wall, along the midclavicular line, approximately 5–6 cm distal to the clavicle. The use of musculoskeletal US in approaching the Pmin ensures accurate placement of the toxin at the target site and helps prevent pneumothorax and injury to the underlying neurovascular bundle [38].

### 2.6. Deltoid Muscle

#### 2.6.1. Overview

The deltoid is a muscle targeted in spastic patterns of the upper limb that involve resistance to passive movement during various maneuvers. These include adduction of the arm, involving all three parts of the muscle; extension and lateral rotation of the arm, which are specific to the clavicular part; adduction of the arm up to 15 degrees, corresponding to the acromial part; and abduction, flexion, and internal rotation of the arm, associated with the spinal part [12,39].

#### 2.6.2. The Clavicular Part of the Deltoid Muscle

##### Ultrasound Identification

The clavicular part of the deltoid, or the anterior deltoid (AD), can be identified using musculoskeletal US by placing the transducer transversely on the anterior surface of the proximal arm, approximately 2–3 cm distal to the clavicle. Superficial to the cortical bone of the humerus, the following structures are observed: the tendon of the long head of the biceps brachii (LHBT), with the lateral and anterior deltoid located superficially to it [34].

##### Key Ultrasound Landmarks

**Figure 8:**
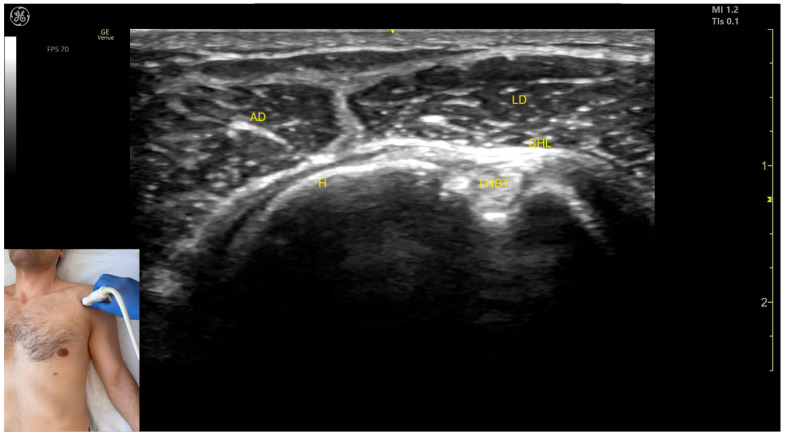
Ultrasound anatomy of the anterior deltoid (AD) with key landmarks: AD—anterior deltoid; LD—lateral deltoid; H—humerus; LHBT—long head bicep tendon; and CHL—coracohumeral ligament.

The key ultrasound features include the following (Figure 8) [8,24,40]:

- Muscle position: It represents a superficial muscle mass on the anterior surface of the proximal arm. Deep to the anterior deltoid, the LHBT is located in the bicipital groove of the humerus, encased in the superficial portion of the coracohumeral ligament (CHL). Superficial to the LHBT, the lateral deltoid is observed, with the AD positioned anterior to the lateral deltoid.
- External fascia: The AD has a pronounced fascia that separates it from the subscapularis tendon, the lateral deltoid, the clavicular head of the pectoralis major, and the subcutaneous muscular plane during BoNT-A injections.
- Dynamic evaluation: During dynamic evaluation, scanning distally toward the elbow joint shows a decrease in the muscle size of the deltoid, coinciding with an increase in the muscle size of the biceps brachii. Scanning proximally toward the clavicle reveals the origin of the AD on the clavicle, along with the appearance and increased size of the clavicular head of the Pmaj [41]. Contraction of the anterior deltoid is seen during flexion and medial rotation maneuvers of the arm at the shoulder joint [12,39].

#### 2.6.3. The Acromial Part of the Deltoid Muscle

##### Ultrasound Identification

The acromial part of the deltoid muscle, or the lateral deltoid (LD), can be identified using musculoskeletal US by placing the transducer transversely on the anterolateral portion of the proximal arm, approximately 2–3 cm distal to the acromion. Superficial to the cortical bone of the humerus, the LD muscle is visualized [40,42].

##### Key Ultrasound Landmarks

**Figure 9:**
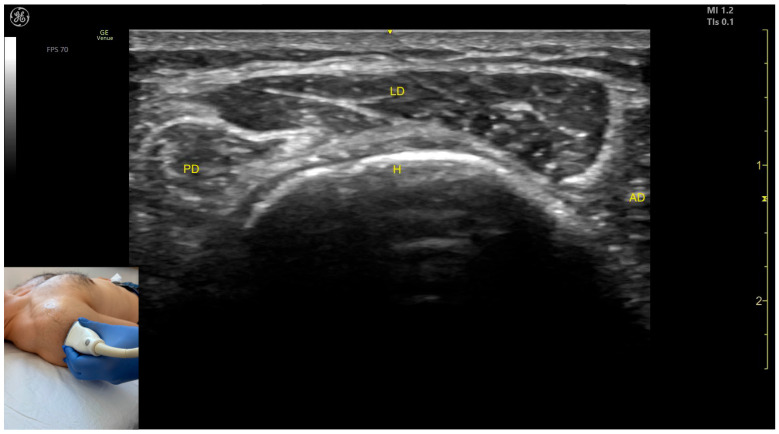
Ultrasound anatomy of the lateral deltoid (LD) with key landmarks: PD—posterior deltoid; LD—lateral deltoid; AD—anterior deltoid; and H—humerus.

The key ultrasound features include the following (Figure 9) [40,42]:

- Muscle position: It represents a superficial muscle mass on the anterolateral surface of the proximal arm. The AD is observed anterior to the LD, while the posterior deltoid is observed posterior to it.
- External fascia: The LD has a pronounced fascia that separates it from the anterior and posterior deltoid parts, as well as from the subcutaneous plane during BoNT-A injections.
- Dynamic evaluation: During dynamic evaluation, proximal scanning toward the acromion reveals the LD originating from the acromion [41]. Dynamic assessment also shows visible contraction of the LD during arm abduction [12,39].

#### 2.6.4. The Spinal Part of the Deltoid Muscle

##### Ultrasound Identification

The spinal part of the deltoid muscle, or the posterior deltoid (PD), can be identified using musculoskeletal US by placing the transducer transversely on the posterior surface of the proximal arm, approximately 2 cm distal to the spine of the scapula. Superficial to the cortical bone of the humerus, the PD and the infraspinatus muscle are visualized [40,42].

##### Key Ultrasound Landmarks

**Figure 10:**
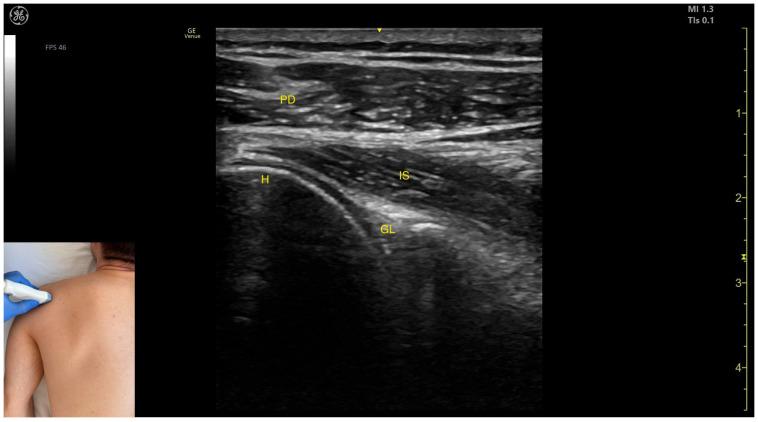
Ultrasound anatomy of the posterior deltoid (PD) with key landmarks: PD—posterior deltoid; IS—infraspinatus; GL—glenoid labrum; and H—humerus.

The key ultrasound features include the following (Figure 10):

- Muscle position: It represents a superficial muscle mass on the posterior surface of the proximal arm. Deep to the PD, the infraspinatus muscle and the glenoid labrum are observed.
- External fascia: The PD has a pronounced fascia that separates it from the infraspinatus muscle and the subcutaneous plane during BoNT-A injections.
- Dynamic evaluation: During dynamic evaluation, the origin of the PD at the spine of the scapula is seen when scanning proximally [41]. Contraction of the PD is visible during dynamic assessment when performing maneuvers involving adduction, extension, and lateral rotation of the arm at the shoulder joint [12,39].

#### 2.6.5. Clinical Implications and Injection Strategy

The deltoid muscle plays a crucial role in shoulder mobility and is commonly involved in spastic patterns affecting the upper limb. According to a 2016 study, local BoNT-A injections into the deltoid muscle can be used in pediatric cerebral palsy due to local spasticity, which leads to significant muscle contractures and deformities of the shoulder joint [43]. In cases where there is resistance to passive movement during adduction of the arm between 15 and 90 degrees, all three parts of the deltoid muscle are injected [44]. In spastic patterns involving abduction and extension of the shoulder joint, the posterior deltoid is the preferred injection site, often in combination with the teres major and latissimus dorsi [11,45]. According to a study by Sheenan et al., in spastic patterns that include shoulder extension, the posterior deltoid is the most frequently injected muscle (75.76%; n = 25), followed by the long head of the triceps brachii (66.67%; n = 22) and the latissimus dorsi (48.48%; n = 16) [25].

BoNT-A injections into the deltoid muscle are also used in pain management for conditions such as muscle spasms and focal dystonia. However, due to insufficient data in the specialized literature regarding optimal injection sites for the three parts of the deltoid, additional studies are necessary [46,47].

In our clinical practice, the preferred injection sites for local BoNT-A administration are at the points of maximum muscle thickness identified via US:

- For the AD, the transducer is placed transversely on the anterior surface of the proximal arm, approximately 2–3 cm distal to the clavicle.
- For the LD, the transducer is positioned transversely on the anterolateral portion of the proximal arm, approximately 2–3 cm distal to the acromion.
- For the PD, the transducer is placed transversely on the posterior surface of the proximal arm, approximately 2 cm distal to the spine of the scapula.

### 2.7. Triceps Brachii (TB)

#### 2.7.1. Overview

The triceps brachii (TB) is a muscle commonly targeted in spastic patterns of the upper limb that involve resistance to passive movement during the following maneuvers [12]:

- Flexion of the forearm at the elbow joint (involving all three heads of the muscle);
- Abduction and flexion of the arm at the shoulder joint (specific to the long head).

#### 2.7.2. Ultrasound Identification

The TB muscle can be identified using musculoskeletal US by placing the transducer transversely at the midpoint of the posterior surface of the upper arm. Superficial and medial to the cortical bone of the humerus, the TB is visualized.

#### 2.7.3. Key Ultrasound Landmarks

**Figure 11:**
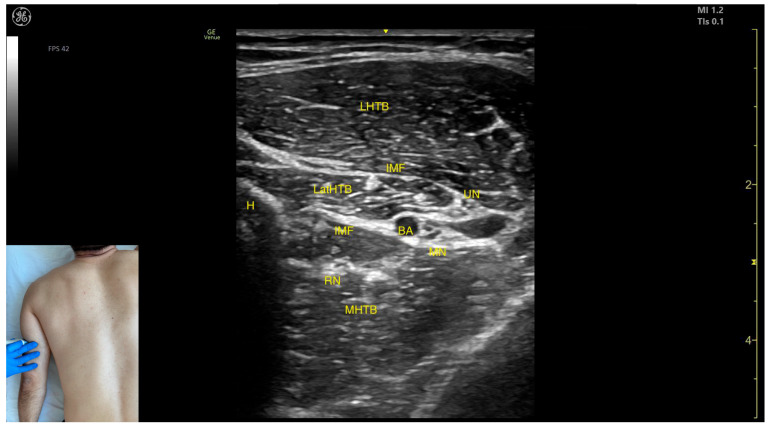
Ultrasound anatomy of the triceps brachii (TB) with key landmarks: LHTB—long head triceps brachii; IMF—intramuscular fascia; LatHTB—lateral head triceps brachii; MHTB—medial head triceps brachii; UN—ulnar nerve; RN—radial nerve; MN—median nerve; BA—brachial artery; and H—humerus.

The key ultrasound features include the following (Figure 11) [48,49]:

- Muscle position: At this level, it represents the only muscle mass in the posterior compartment of the arm.
- Muscle morphology: The TB has three heads—long, medial, and lateral—each of which can be approached individually. These three heads are separated by intramuscular fasciae:
  1. Long head (LHTB): Located superficial to the cortical bone of the humerus.
  2. Medial head (MHTB): Found medial to the humeral cortex.
  3. Lateral head (LatHTB): Situated between the long and medial heads.
- Innervation and vascular supply: In the medial portion of the TB, the neurovascular bundle is observed, consisting of three nerves—median, radial, and ulnar—and two vascular structures, namely the brachial artery with its branches and the brachial vein.
- External fascia: The TB features a pronounced fascia that separates it from the superficial plane during BoNT-A injections.
- Dynamic evaluation: During dynamic evaluation, scanning distally toward the elbow joint reveals a decrease in the size of the long and lateral heads, while the medial head shows an increase in size. Muscle contraction is visible during elbow extension maneuvers, confirming its role in this movement [12,50].

#### 2.7.4. Clinical Implications and Injection Strategy

According to a 2023 study, in spastic patterns involving shoulder extension, the TB is a frequently injected muscle with BoNT-A, second only to the posterior deltoid [25]. For biceps–triceps co-contraction, multiple specialized studies have demonstrated that TB injection effectively reduces resistance to passive elbow flexion [51,52,53].

A 2023 study by Yi et al. identified the zones with the highest density of intramuscular nerve arborization in the TB, with the densest areas located at 30–50% and 60–70% for the long head, 30–40% for the lateral head, and 30–60% for the medial head, along a reference line drawn from the midpoint of the olecranon (0%) to the anteroinferior point of the acromion [54].

In our clinical practice, the preferred injection site for BoNT-A is at the point of maximum muscle thickness identified via musculoskeletal US, with the transducer placed transversely on the posterior surface of the arm.

- Long head: Injected at two sites—one at the midpoint of the posterior arm and another 2 cm proximal to this level, closer to the shoulder joint.
- Lateral head: Injected at the midpoint of the posterior arm.
- Medial head: Injected 1 cm distal to the midpoint of the posterior arm.

### 2.8. Biceps Brachii (BB)

#### 2.8.1. Overview

The biceps brachii (BB) is a muscle frequently targeted in spastic patterns of the upper limb that involve resistance to passive movement during elbow extension, forearm pronation, and shoulder extension [12,55].

#### 2.8.2. Ultrasound Identification

The BB muscle can be identified using musculoskeletal US by placing the transducer transversely on the anterior surface of the arm, at the distal third. Superficial to the cortical bone of the humerus, two oval-shaped muscle masses are visualized: the brachialis and the BB [56].

#### 2.8.3. Key Ultrasound Landmarks

**Figure 12:**
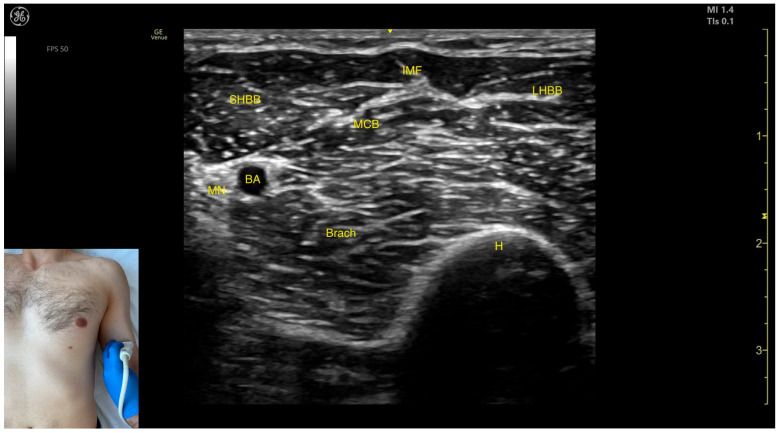
Ultrasound anatomy of the biceps brachii (BB) with key landmarks: SHBB—short head biceps brachii; LHBB—long head biceps brachii; IMF—intramuscular fascia; MCB—musculocutaneous branch; MN—median nerve; BA—brachial artery; Brach—brachialis; and H—humerus.

**Figure 13:**
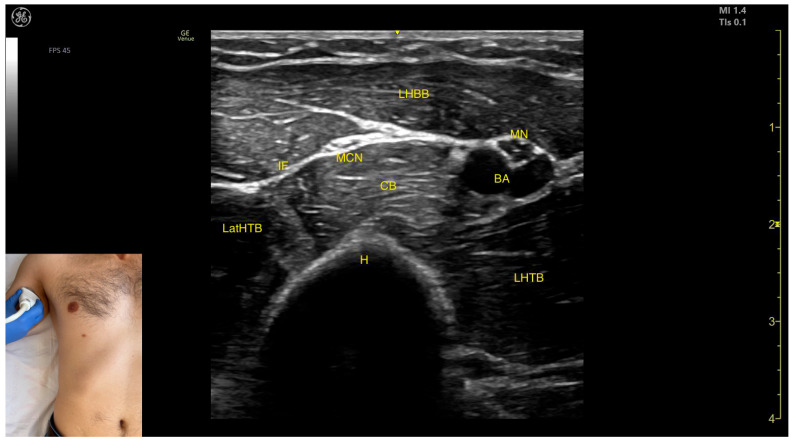
Ultrasound anatomy of the long head biceps brachii (LHBB) with key landmarks: LHBB—long head biceps brachii; IF—intermuscular fascia; MCN—musculocutaneous nerve; CB—coracobrachialis; MN—median nerve; BA—brachial artery; LatHTB—lateral head triceps brachii; H—humerus; and LHTB—long head triceps brachii.

The key ultrasound features include the following (Figure 12 and Figure 13) [56,57]:

- Muscle position: It represents the most superficial muscle mass of the anterior arm at this level [57].
- Muscle morphology: It has two heads, with the long head (LHBB) located laterally and the short head (SHBB) positioned medially, separated by an intramuscular fascia that allows them to be individually targeted during procedures.
- Innervation and vascular supply: In the depth of the muscle at the level of the bicipital fossa (medial portion), the brachial artery is observed laterally, while the median nerve is located medially to the artery, with the bicipital fossa representing a longitudinal groove bordered anteriorly by the BB and brachialis muscles and posteriorly by the medial head of the triceps brachii.
- External fascia: The BB lacks a pronounced fascia that separates it from the brachialis, which is relevant when performing BoNT-A injections.
- Dynamic evaluation: During dynamic evaluation (Video S1), proximal scanning toward the shoulder joint reveals a decrease in the size of the brachialis muscle until it transforms into a tendon at the mid-arm level, where the insertion of the coracobrachialis (CB) muscle on the humerus can be observed deep to the BB. Scanning further toward the proximal third of the arm shows the inversion of the positions of the median nerve and brachial artery within the bicipital fossa [57]. Continuing the scan proximally toward the shoulder joint reveals an increase in the size of the CB muscle in the medial portion of the arm. The musculocutaneous nerve is usually seen piercing the coracobrachialis before descending in the intermuscular fascia (IF) between the BB and brachialis. The CB muscle is not injected with BoNT-A due to its minor role as a weak flexor of the arm at the shoulder joint. The musculocutaneous nerve is targeted during diagnostic nerve block procedures to differentiate between spasticity and rigidity in cases where severe spasticity restricts elbow flexion or extension. Branches of the musculocutaneous nerve can be visualized via US on the anterior surface of the BB, appearing prominently and potentially being mistaken for intramuscular fascia [58]. The contraction of the BB can be observed during flexion maneuvers of the elbow and shoulder joints. With the elbow flexed at 90 degrees, performing forearm supination shows the BB gliding over the underlying brachialis muscle (Video S2) [12,55].

#### 2.8.4. Clinical Implications and Injection Strategy

According to a study by Moon et al., which aimed to determine the motor points of the BB muscle using EMG, the highest concentration of motor points for both the short head and long head of the BB was identified at the distal third of the arm, in the medial and lateral portions, respectively [59].

In our clinical practice, the preferred injection site for local BoNT-A administration is at the point of maximum muscle thickness, identified via musculoskeletal US. The transducer is placed transversely on the anterior surface of the arm at the distal third.

- At this level, the short head is injected into the medial portion;
- The long head is injected into the lateral portion.

### 2.9. Brachialis Muscle (Brach)

#### 2.9.1. Overview

The brachialis muscle (Brach) is commonly targeted in spastic patterns of the upper limb that involve resistance to passive movement during forearm extension at the elbow joint [7].

#### 2.9.2. Ultrasound Identification

The Brach can be identified using musculoskeletal US by placing the transducer transversely on the anterior surface of the arm at the distal third, specifically in the lateral portion. Superficial to the cortical bone of the humerus, the Brach muscle is visualized [60].

#### 2.9.3. Key Anatomical Landmarks

**Figure 14:**
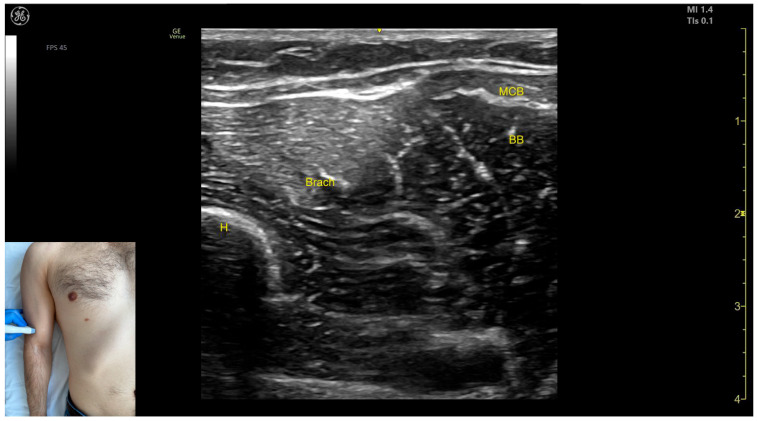
Ultrasound anatomy of the brachialis (Brach) with key landmarks: Brach—brachialis; H—humerus; MCB—musculocutaneous branch; and BB—bicep brachii.

The key ultrasound features include the following (Figure 14) [60,61]:

- Muscle position: It represents the first muscle mass located superficial to the cortical bone of the humerus on the anterior lateral surface of the arm.
- Innervation: The radial nerve is seen superficially in the intermuscular fascia, which separates the Brach from the brachioradialis muscle.
- External fascia: The Brach has a pronounced fascia that separates it from the brachioradialis. A very thin fascia separates it from the BB during BoNT-A injections.
- Dynamic evaluation: During dynamic evaluation, proximal scanning toward the shoulder joint shows the radial nerve moving closer to the cortical bone of the humerus before following an oblique path and disappearing from the view as it enters the posterior compartment of the arm. Medial scanning reveals the BB muscle superficial to the Brach. Muscle contraction of the Brach is visible during forearm flexion at the elbow joint, as it is the only pure flexor of the elbow [7].

#### 2.9.4. Clinical Implications and Injection Strategy

The zone with the highest density of intramuscular nerve branches corresponding to the Brach is located at the distal third of the arm [62].

In our clinical practice, the preferred injection site for local BoNT-A administration is at the point of maximum muscle thickness identified via musculoskeletal US. The transducer is placed transversely on the anterolateral surface of the arm at the distal third, approximately 4–6 cm proximal to the lateral epicondyle, where the radial nerve is positioned along the humerus.

- Medial to the radial nerve, the zone of maximum thickness of the Brach is visualized.
- Medial to the Brach at this level lies the BB, which can be dynamically confirmed by performing a forearm supination maneuver (Video S3).

This ultrasound-guided approach ensures accurate targeting of the Brach while minimizing the risk of radial nerve injury during the procedure.

### 2.10. Brachioradialis (BR)

#### 2.10.1. Overview

The brachioradialis (BR) is a muscle commonly targeted in spastic patterns of the upper limb that involve resistance to passive movement during elbow extension, particularly when the forearm is positioned midway between supination and pronation [12].

#### 2.10.2. The Arm Compartment

##### Ultrasound Identification

The BR can be identified using musculoskeletal US by placing the transducer transversely on the anterolateral surface of the arm, approximately 2–3 cm proximal to the lateral epicondyle. Superficial to the cortical bone of the radius, two muscle masses are visualized: the Brach muscle (deep) and the BR muscle (superficial).

##### Key Anatomical Landmarks

**Figure 15:**
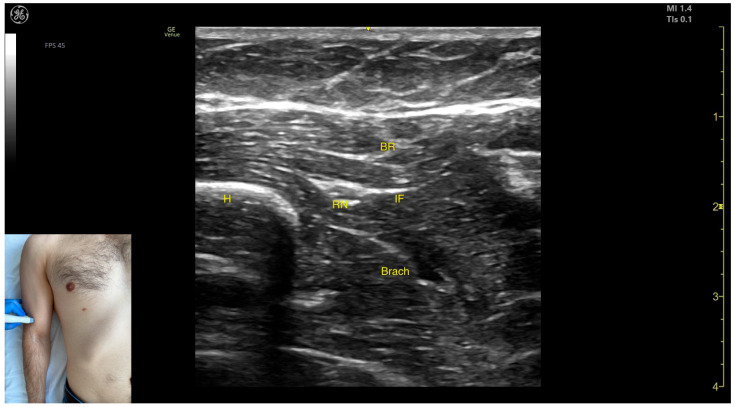
Ultrasound anatomy of the brachioradialis (BR) in the arm compartment with key landmarks: H—humerus; BR—brachioradialis; RN—radial nerve; IF—intermuscular fascia; and Brach—brachialis.

The key ultrasound features include the following (Figure 15) [61,63,64]:

- Muscle position: It represents the most superficial muscle mass on the anterolateral surface of the arm at this level.
- Innervation: The radial nerve is located deep within the muscle, enclosed in the intermuscular fascia that separates the BR from the Brach muscle.
- External fascia: The BR has a pronounced fascia that separates it from both the Brach and the subcutaneous plane during BoNT-A injections.
- Dynamic evaluation: During dynamic evaluation, distal scanning toward the lateral epicondyle reveals the division of the radial nerve into its superficial branch and deep branch (posterior interosseous nerve), with the deep branch continuing along the anterolateral proximal third of the forearm.

#### 2.10.3. The Forearm Compartment

##### Ultrasound Identification

The BR muscle can be identified using musculoskeletal US by placing the transducer transversely on the anterolateral surface of the forearm, approximately 2–3 cm distal to the lateral epicondyle. From deep to superficial, the cortical bone of the radius is visualized, along with four muscles: the supinator (with the posterior interosseous nerve located between its heads), the extensor carpi radialis brevis, the extensor carpi radialis longus, and the BR.

##### Key Anatomical Landmarks

**Figure 16:**
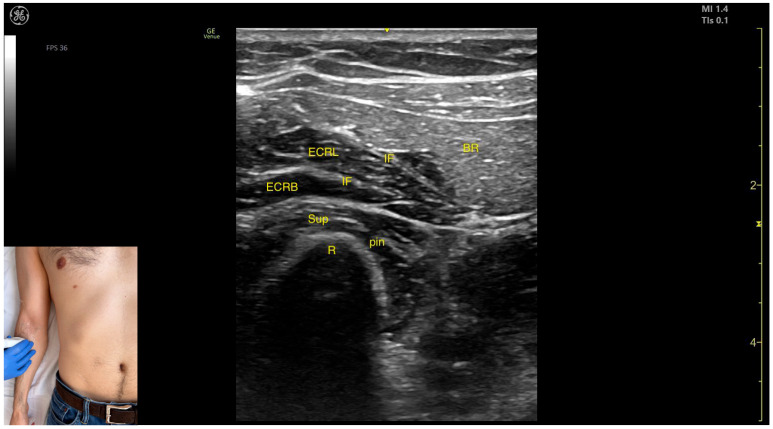
Ultrasound anatomy of the brachioradialis (BR) in the forearm compartment with key landmarks: BR—brachioradialis; IF—intermuscular fascia; ECRL—extensor carpi radialis longus; ECRB—extensor carpi radialis brevis; Sup—supinator; R—radius; and pin—posterior interosseous nerve.

The key ultrasound features include the following (Figure 16) [12,19,63]:

- Muscle position: It represents the most superficial muscle mass at this level.
- External fascia: It has a thin intermuscular fascia that separates it from the extensor carpi radialis longus muscle, located deep to it.
- Dynamic evaluation: During dynamic evaluation, the BR can be differentiated from the extensor carpi radialis longus by performing wrist extension and abduction maneuvers with the forearm in pronation, during which contraction of the extensor carpi radialis longus is observed (Video S4). Contraction is visible both in the arm and forearm during elbow extension maneuvers, with the forearm positioned midway between supination and pronation.

#### 2.10.4. Clinical Implications and Injection Strategy

A 2017 study by Yang et al. identified the intramuscular regions with the highest nerve density in the BR muscle as being located at 39.04–61.90% and 73.80–90.47% of the muscle length, measured from origin to insertion [65].

In our clinical practice, the preferred injection site for local BoNT-A administration is at the point of maximum muscle thickness identified via musculoskeletal US, using the following two approaches:

- The transducer is placed transversely on the anterolateral surface of the arm, approximately 2–3 cm proximal to the lateral epicondyle.
- The transducer is placed transversely on the anterolateral surface of the forearm, approximately 2–3 cm distal to the lateral epicondyle.

The use of musculoskeletal US in approaching the BR muscle ensures precise placement of the toxin at the target site and helps to avoid nerve injury. However, for less experienced personnel, the intermuscular fascia separating the BR from the extensor carpi radialis longus may present a potential source of error when injecting the BR in the forearm compartment.

To differentiate the BR from the extensor carpi radialis longus, a wrist extension and abduction maneuver is performed with the forearm in pronation (Video S4) [19].

## 3. Conclusions

This visual guide complements the previously published part focusing on distal upper limb muscles, offering a complete reference for the ultrasound-guided management of upper limb spasticity. By synthesizing data from the literature and sharing our clinical experience at the Elias University Hospital (EUH), we aim to help clinicians achieve precise botulinum toxin type A (BoNT-A) delivery, minimize complications, and optimize functional outcomes.

The detailed anatomical insights, ultrasound identification techniques, and clinical implications, along with injection strategies presented for each proximal upper limb muscle, establish a robust evidence-based framework for enhancing patient care in spasticity management. This guide incorporates extensive high-quality images and videos, providing clinicians with visual support to refine their injection techniques and ensure accurate BoNT-A administration. Through this systematic approach, the authors aim to standardize ultrasound-guided injection techniques, improve clinical outcomes, and advance the field of post-stroke spasticity management.

## Supplementary Materials

The following supporting information can be downloaded at: https://doi.org/10.5281/zenodo.14996875 (accessed on 9 March 2025). Video S1: Ultrasonographic evaluation of the musculocutaneous nerve (MCN); Video S2: Dynamic ultrasonographic evaluation of the biceps brachialis (BB); Video S3: Anatomical and dynamic evaluation of elbow flexors; Video S4: Dynamic evaluation of the brachialis muscle (Brach) in the forearm compartment.

## Abbreviations

The following abbreviations are used in this manuscript:

BoNT-A	botulinum toxin type A
EUH	Elias University Hospital
LD	latissimus dorsi (muscle)
SA	serratus anterior (muscle)
ICM	intercostal muscle
P	pleura
Rb	rib
TM	teres major (muscle)
Tm	teres minor (muscle)
SSC	subscapularis (muscle)
Scap	scapula
US	ultrasound
IMFS	intramuscular fascial septum
LHBT	long head of the biceps brachii tendon
CHL	coracohumeral ligament
AD	anterior deltoid (muscle)
SSC-T	subscapularis tendon
Pmaj	pectoralis major (muscle)
Pmin	pectoralis minor (muscle)
PMC	clavicular head of the pectoralis major (muscle)
PMS	sternocostal head of the pectoralis major (muscle)
IMF	intramuscular fascia
n	nerve
A	artery
V	vein
ICM	intercostal muscle
LD	lateral deltoid (muscle)
PD	posterior deltoid (muscle)
GL	glenoid labrum
TB	triceps brachii (muscle)
LHTB	long head of the triceps brachii (muscle)
MHTB	medial head of the triceps brachii (muscle)
LatHTB	lateral head of the triceps brachii (muscle)
BA	brachial artery
MN	median nerve
RN	radial nerve
UN	ulnar nerve
BB	biceps brachii (muscle)
SHBB	short head of the biceps brachii (muscle)
LHBB	long head of the biceps brachii (muscle)
CB	coracobrachialis (muscle)
MCB	musculocutaneous branch (nerve)
Brach	brachialis (muscle)
IF	intermuscular fascia
EMG	electromyography
BR	brachioradialis (muscle)
ECRL	extensor carpi radialis longus (muscle)
ECRB	extensor carpi radialis brevis (muscle)
Sup	supinator (muscle)
pin	posterior interosseous nerve
H	humerus
